# Visual grading of valvular regurgitation is inferior to measurement – results from the VIAVA-study (VIsual Assessment of VAlvular Regurgitation)

**DOI:** 10.1186/s44156-024-00061-0

**Published:** 2024-11-11

**Authors:** Ozan Demirel, Paolo Di Stefano, Elke Boxhammer, Thomas Wuppinger, Christina Granitz, Björn Goebel, Uta C. Hoppe, Michael Lichtenauer, Moritz Mirna

**Affiliations:** 1 Clinic of Internal Medicine II, Department of Cardiology, Paracelsus Medical University of Salzburg, Müllner Hauptstraße 48, Salzburg, 5020 Austria; 2 Department of Cardiology, Heart Center of the Central Clinic Bad Berka, Bad Berka, 99438 Germany

**Keywords:** Echocardiography, Valvular Regurgitation, Visual Estimation, Quantitative Assessment

## Abstract

While the visual estimation of systolic left ventricular function by experienced examiners closely aligns with quantitative methodologies, the accuracy of visual estimation in determining the severity of valvular regurgitation using colour flow Doppler assessment of native heart valves remains largely unexplored. This study analysed the ability of 262 physicians to visually estimate the severity of 12 native valve regurgitations by grading colour Doppler transthoracic echocardiography loops in an online questionnaire. The assessments of the participants were compared to standardized quantitative evaluations conducted by certified echocardiography experts. Of the three valves to assess, evaluations by the participants showed the best correlation (Rs = 0.75, *p* < 0.0001) and agreement (percent agreement: 66.4%) with those of the experts in mitral valve regurgitation (MR). High agreement was observed for mild regurgitation across all valves (MR 94.5%, AR 80.3% and TR 88.7%), while consensus diminished in moderate (MR 55.9%, AR 49.5% and TR 55.0%) and severe regurgitation (MR 57.6%, AR 67.4%, TR 14.6%). The study underscores the potential utility of visual estimation of valvular regurgitation in clinical settings for identifying clinically relevant regurgitations. However, our findings also highlight the importance of integrating visual estimation with quantitative methods, particularly in moderate and severe cases of regurgitation.

**Supplementary Information:**

The online version contains supplementary material available at 10.1186/s44156-024-00061-0.

## Background

In recent decades, technological innovation has progressed at an astonishing rate, resulting in increasingly compact and inexpensive electronic devices [1]. Owing to the constant enhancement of technical capabilities and widespread availability of ultrasound devices, transthoracic echocardiography (TTE) has become a standard examination in internal medicine. Using “handheld ultrasound”, it is now possible to perform examinations at the patient’s bedside and even using continuous wave (CW) Doppler analysis [2–4]. When used by trained cardiologists, these devices have a high sensitivity in detecting and assessing clinically relevant valvular regurgitation in hospitalized patients [5]. The structural and functional insights gained from a correctly performed examination are comprehensive and assist the physician in addressing numerous diagnostic and therapeutic decisions in daily practice. The accessibility of TTE and the information it provides means that it can be applied in a variety of settings, from hospital emergency departments to private practices [6].

Despite the advancements made, the field faces challenges in maintaining consistency in the quality of echocardiographic findings, particularly with the increasing use of advanced analysis methods such as three-dimensional (3D) echocardiography and strain analyses [7, 8]. This ultimately resulted in efforts to standardize findings internationally, based on defined criteria and measured values [9, 10]. Valvular disease should be graded by TTE using a combination of several morphological and quantitative criteria (e.g. vena contracta, proximal isovelocity surface area (PISA), regurgitation fraction and volume, ventricular function, ventricular and atrial size, etc.), according to the current recommendations of the European Association of Cardiovascular Imaging (EACVI) [11] and the guidelines of the European Society of Cardiology (ESC) [12]. However, in clinical practice, assessing valvular regurgitation comprehensively using all technically feasible measurements and criteria is often limited due to various factors such as lack of qualification, knowledge, or time [13].

While colour flow Doppler assessment can provide simple estimates of valvular regurgitation severity, it has many limitations due to variable factors like hemodynamics and gain settings [14]. Therefore, it is not recommended for the quantitative assessment of aortic valve regurgitation (AR), mitral valve regurgitation (MR), or tricuspid valve regurgitation (TR) severity [11]. Nevertheless, while visual estimation of systolic left ventricular function (LVF) by experienced examiners is in close agreement with quantitative methods [15–18], the agreement of visual estimation and the extent of valvular regurgitation in native heart valves has not been thoroughly investigated yet. The aim of this study is to evaluate the agreement of visual estimation (also known as ‘eyeballing’) of valvular regurgitation compared with the currently recommended comprehensive standardised quantitative approach.

## Materials and methods

The study was performed in compliance with the principles of Good Clinical Practice and the Declaration of Helsinki. The ethics committee of the state of Salzburg was informed prior to data collection and gave consent that no formal approval by the committee was necessary (415-EALL/4/152/5-2022).

### Participants

Participants were recruited through an online survey distributed to 4481 email addresses of internal medicine departments and doctors working in internal medicine departments across Germany (*n* = 3155), Austria (*n* = 1229), and Switzerland (*n* = 97) from August 2023 to January 2024. The online survey was created using the Qualtrics CoreXMplatform (Qualtrics, Provo, United States; accessed November 6th 2022) and was accessible via computer or smartphone (see Supplementary Fig. S2).

### Selection of TTE-loops

TTE datasets were selected from patients treated at the Department of Internal Medicine II at the University Hospital of Salzburg, Austria, between July 2021 and May 2023. Twelve cases of valve regurgitation were included, with four cases each of aortic, mitral, and tricuspid valve regurgitation, and varying degrees of severity ranging from grade 1 (mild) to grade 3 (severe). Two EACVI TTE certified echocardiography experts (C.G. and T.W.) rigorously assessed each case using formal and quantitative criteria in accordance with the current recommendations of the ESC and EACVI [11, 12]. The experts had access to the full TTE dataset (see Supplementary Table 1). Participants in the study were able to assess each valvular regurgitation only with colour flow Doppler TTE recordings in two planes. The images and loops were anonymized to protect patient identity.

#### Primary and secondary outcome measures

The primary outcome measure of this study was percent agreement of the grades attributed by participants with the ratings of the experts in regard to each valve. Secondary outcome measures were interrater agreement between participants, correlation with the expert ratings and statistical differences in the absolute number of correct assessments between subgroups.

### Statistical analysis

Statistical analysis was conducted with R (version 4.2.1., R Core Team (2013), R Foundation for Statistical Computing, Vienna, Austria; http://www.R-project.org/) using the packages ‘Rcmdr’, ‘ggplot2’, ‘pastecs’, ‘Hmisc’, ‘ggm’, ‘polycor’, ‘QuantPsyc’, ‘glmnet’, ‘psych’ and ‘irr’ and SPSS (Version 29.0, IBM, Armonk, New York, USA). Categorical data were assessed with Fisher’s exact test. Distribution of continuous data was assessed by Kolmogorov Smirnov test and visually. Since data distribution was not normal, medians between subgroups were compared using Kruskal Wallis test with Dunn’s post hoc test. Spearman’s rank correlation coefficient was used for correlation analyses. Interrater agreement was assessed by Kendall’s coefficient of correlation. The association between baseline covariates and score of correct assessments was investigated using univariate linear regression analysis. Prior, continuous data were transformed into z-scores and the distribution of residuals as well as the presence of homoscedasticity were checked by histogram and scatterplot. A p-value of < 0.05 was considered statistically significant.

## Results

In total, 262 persons participated in the study. Baseline characteristics of the participants are depicted in Table 1.

In brief, 61.9% (*n* = 161) were male and the median age of the participants was 35 years (IQR 31–42). Most participants were working in a hospital of 3rd level of care (39.3%, *n* = 103) and most participants were resident physicians (40.2%, *n* = 104; see Table 1).

**Table 1:** Baseline covariates of the participants. *Abbreviations*: IQR = interquartile range, Hosp. 1st level = hospital of first level of care, Rehab.= rehabilitation center, TTE = transthoracic echocardiography

	Median	IQR				
Age (years)	35	31–42				
Experience (years)	5	2–10				
Exams per day (n)	4	2–10				
	Male	Female	Non-binary/other	Not specified		
Gender (%)	61.9 (161)	36.9 (96)	0.4 (1)	0.8 (2)		
	Austria	Germany	Switzerland	Italy	Princ. of Liechtenstein	
Country (%)	53.3 (138)	39.8 (103)	6.2 (16)	0.4 (1)	0.4 (1)	
	Hands-on course	Specialist Cardiology	Specialist Internal medicine	TTE-diploma by society	Other (e.g. Online course)	
Education (%)	58.8 (147)	34.8 (87)	33.2 (83)	20.2 (50)	1.5 (2)	
	Hosp. 3rd level	Hosp. 2nd level	Hosp. 1st level	Doctor’s office	Other (e.g. Rehab. center)	
Place of work (%)	39.3 (103)	32.4 (85)	22.9 (60)	1.9 (5)	3.4 (9)	
	Resident	Senior Physician	Specialist	Head Physician	Intern	Medical Student
Position (%)	40.2 (104)	35.9 (93)	17.0 (44)	3.5 (9)	1.9 (5)	1.5 (4)

Concerning education in the field of TTE, most participants stated that they had a hands-on echo course (58.8%, *n* = 147), whereas a TTE-diploma issued by a society was present in only 20.2% (*n* = 50). Residents more often stated that they had a hands-on echo course than specialists, senior physicians or head physicians (77.1% (*n* = 74) vs. 51.2% (*n* = 22) vs. 48.4% (*n* = 45) vs. 11.1% (*n* = 1), *p* < 0.0001), while a diploma was more often present in senior physicians or specialists (residents: 9.4% (*n* = 9) vs. senior physicians: 29.7% (*n* = 27) vs. specialists: 27.9% (*n* = 12), *p* = 0.004; see Table 1).

In regard to experience in the field of TTE, participants regularly conducted TTEs since a median of 5 years (IQR 2–10) and the median number of examinations during a normal working day was 4 (IQR 2–10; see Table 1).

### Primary and secondary outcome measures per valve

**Fig. 1:**
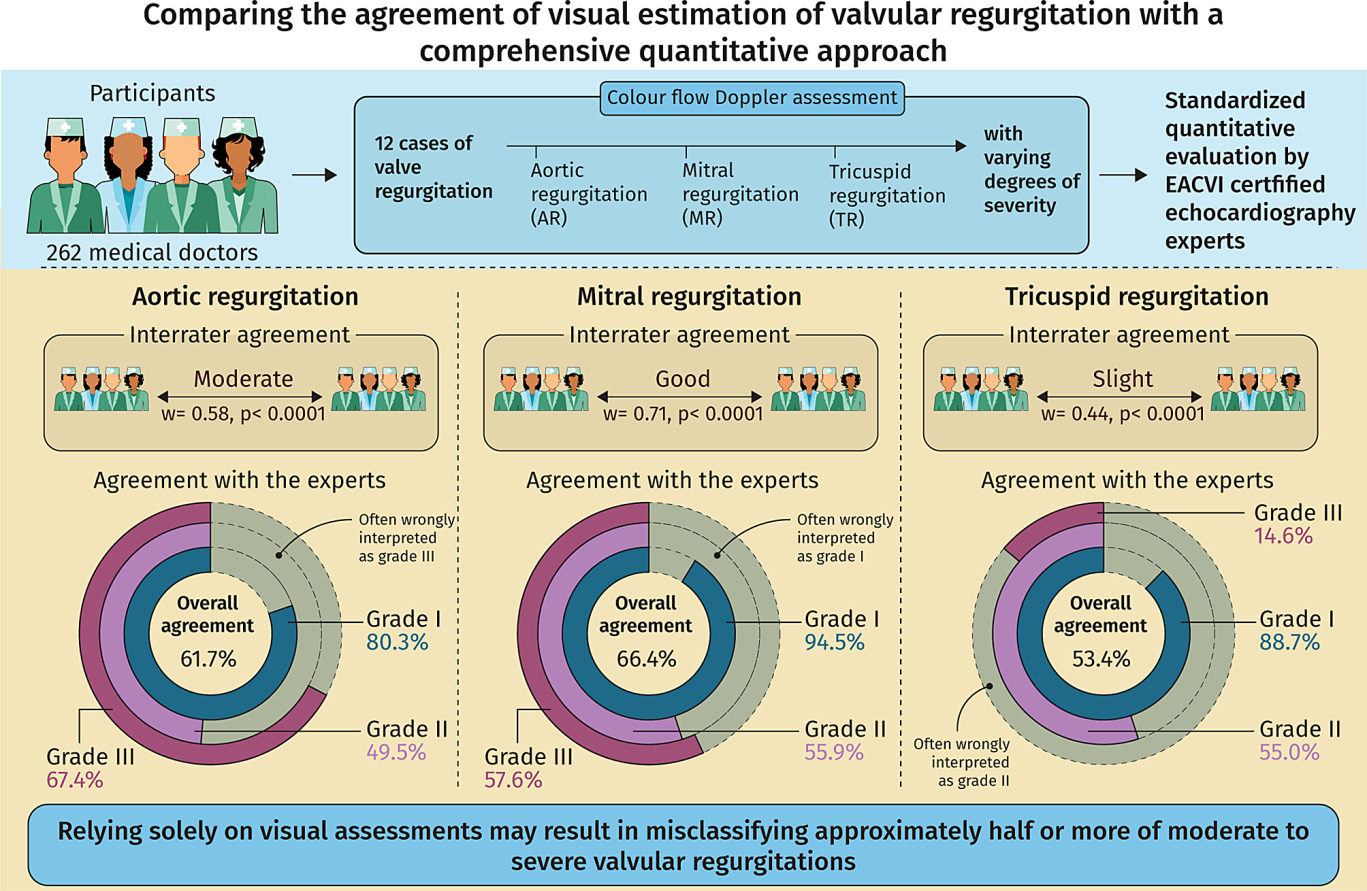
Graphical summary of the VIAVA-study. *Abbreviations*: AR = aortic valve regurgitation, MR = mitral valve regurgitation, TR = tricuspid valve regurgitation

Regarding the four cases of AR (1 grade I (mild), 2 grade II (moderate), 1 grade III (severe)), a total of 914 assessments by the participants were recorded. Interrater agreement between the participants was moderate, but statistically significant (w = 0.58, *p* < 0.0001; see Fig. 1). Recorded assessments showed a positive correlation with the grades attributed by the experts (Rs = 0.62, *p* < 0.0001), whereas percent agreement with the experts was 61.7%. AR grade I and grade III were most often correctly identified, with grade I correctly identified in 80.3% (*n* = 184), grade II in 49.5% (*n* = 225) and grade III in 67.4% (*n* = 155). In case of an incorrect answer, AR grade II was most often wrongly interpreted as grade III (47.0%, *n* = 214; see Supplementary Fig. 1).

Four cases of MR (1 grade I (mild), 1 grade II (moderate), 2 grade III (severe)) resulted in 874 assessments by the participants. Interrater agreement was good and statistically significant (w = 0.71, *p* < 0.0001; see Fig. 1). Assessments by the participants again showed a positive correlation with the grades by the experts (Rs = 0.75, *p* < 0.0001) and percent agreement was 66.4%. Again, MR grade I and III were the cases which were most often correctly classified by the participants. Grade I was correctly identified in 94.5% (*n* = 206) of assessments, grade II in 55.9% (*n* = 123) and grade III in 57.6% (*n* = 251). When participants incorrectly evaluated MR grade II, they predominately interpreted it as a grade I regurgitation (28.2%, *n* = 62; see Supplementary Fig. 1).

For TR, four cases had to be evaluated by participants (1 grade I (mild), 2 grade II (moderate), 1 grade III (severe)) which led to the submission of 847 assessments. In comparison to the other two valves, interrater agreement was slight, yet statistically significant (w = 0.44, *p* < 0.0001; see Fig. 1). Albeit weak, grades by the participants again showed a positive correlation with the grades by the experts (Rs = 0.45, *p* < 0.0001). Percent agreement with the experts was 53.4%. TR grade I and II were most often correctly identified, with grade I correctly evaluated in 88.7% (*n* = 189), grade II in 55.0% (*n* = 232) and grade III in only 14.6% (*n* = 31). When participants incorrectly evaluated TR grade III, they most often wrongly interpreted it as TR grade II (65.1%, *n* = 138; see Supplementary Fig. 1).

### Number of assessments per subgroup

On average, participants graded 57.8% ±18.0 (median: 7, IQR 6–8) of 12 assessed loops correctly. We observed no difference in the number of correctly assessed TTE-loops in regard to the position stated (*p* = 0.926) or the place of work of the participants (*p* = 0.463; see Table 2).

**Table 2:** Number of correct assessments per position or place of work of the participants. *Abbreviations*: IQR = interquartile range

Participant position	Correct assessments (median number)	IQR	*p*-value
- Medical student	8	5–9	0.926
- Intern	7	4–9	
- Resident	7	5–8	
- Specialist	7	5–9	
- Senior physician	7	6–9	
- Head physician	8	6–9	
Place of work	Correct assessments (median number)	IQR	p-value
- Doctor’s office	8	6–8	0.463
- Hospital 1st level	7	6–8	
- Hospital 2nd level	7	6–9	
- Hospital 3rd level	7	5–9	
- Other	6	4–8	

### Linear regression analysis

In order to investigate the association between the absolute number of correct assessments of TTE loops and different predictor variables, we further performed univariate linear regression analysis. Of all possible predictors investigated, only the variable “Specialist Cardiology” was predictive for a better assessment score (β = 0.424, SE = 0.135, *p* = 0.002), whereas “Hands-on course” was negatively associated with the score (β= -0.345, SE = 0.132, *p* = 0.010; see Table 3).

**Table 3:** Univariate linear regression analysis of different covariates with the absolute number of correct assessments. *Abbreviations*: internal med.= internal medicine, TTE = transthoracic echocardiography, years exp.= years of experience

	β	SE	*p*-value	adj. *R*²	F
Participant position (e.g. resident, senior physician)	0.076	0.063	0.230		
Place of work	0.084	0.081	0.297		
Hands-on course	-0.345	0.132	0.010	0.030	6.79
TTE-diploma by society	0.147	0.167	0.381		
Specialist Internal medicine	-0.101	0.140	0.471		
Specialist Cardiology	0.424	0.135	0.002	0.039	9.88
Age, z-score	0.009	0.068	0.889		
Years of experience., z-score	-0.011	0.067	0.867		
Exams per day, z-score	0.039	0.065	0.552		

## Discussion

This study aimed to assess agreement of visual estimation of valvular regurgitation with a comprehensive, standardized quantitative approach. While participants showed high accuracy for mild regurgitations, the reliability of visual estimations decreased for moderate cases and, in the case of TR, was notably poor for severe regurgitation due to underestimation. Higher accuracy was observed in the detection of mainly mild and, to a lesser extent, severe regurgitations. This finding indicates that it is easier to estimate extreme severities than moderate regurgitations. Furthermore, the discrepancy in severe TR could be attributed to the inherent challenges in visualizing the tricuspid valve or the less frequent clinical focus on TR compared to aortic or mitral valve disease.

Our study shows that visual estimation can be helpful in mild regurgitations, but quantitative methods are crucial for accurate diagnosis and proper patient management as the severity of regurgitation increases. Previous research has also highlighted the limitations of estimating valve regurgitation using colour flow Doppler and emphasized the importance of quantitative methods for accurate diagnosis [19–24]. One such limitation was investigated by Losordo et al. in 1993, who assessed the reliability of using colour flow Doppler imaging for estimating regurgitant volume in valvular regurgitation in experimental models [25]. The authors found that the visual estimation of regurgitation severity based on colour Doppler images is influenced more by the velocity of the jet flow rather than its actual volume, challenging the accuracy of this method for quantifying valvular regurgitation. However, our findings add to the literature by quantifying the degree of agreement among a broad range of clinicians.

It is important to acknowledge that even quantitative echocardiographic analysis of MR can occasionally fail to accurately describe hemodynamic conditions. This highlights the necessity of comprehensive and standardized image acquisition for proper assessment [26]. In MR in particular, visual assessment of eccentric jets and severe mitral annulus calcifications with acoustic shadowing can result in an underestimation of MR severity [27]. Consequently, the structural and functional data provided by cardiac magnetic resonance imaging (CMR) are of considerable value, and should be considered in cases where echocardiographic assessment is inconclusive or inconsistent with clinical observations [12, 28, 29]. In cases of AR, CMR allows direct measurement of the regurgitant flow and is also particularly beneficial in the presence of eccentric jets or multiple valve lesions [30, 31]. The precise quantitative assessment of regurgitation and ventricular volumes makes CMR highly valuable in determining the severity of MR and TR [32]. Additionally, CMR can characterise myocardial tissue, identifying fibrosis or scarring, which offers further prognostic information. It is noteworthy that the increasing integration of 4-dimensional (4D) Flow CMR into clinical practice represents a further promising advancement for the future of valve assessment [33].

The observed negative association between participation in a hands-on echocardiography course and the accuracy of regurgitation assessment may be attributed to a number of underlying factors. An overreliance on course certifications may foster a false sense of competence, thereby obscuring the necessity for continuous skill refinement through practical experience. This may indicate a selection bias, where whereby practitioners with less experience are more likely to seek additional training than their more advanced colleagues who rely more on their clinical experience. Thus, while courses are important for learning basic skills, the nuanced skills required for accurate assessments are possibly honed through hands-on experience and continued learning outside of formal training environments.

Our study highlights the significance of utilizing the colour Doppler method as the first step in identifying valve regurgitation. This enables clinicians to differentiate between potentially clinically relevant regurgitation (moderate or severe) and less relevant cases (mild). Subsequently, a comprehensive standardized quantitative approach should be used to meticulously grade moderate to severe regurgitations detected through this method. This method can balance the need for expedient bedside assessments with the importance of accurately grading the severity of valvular regurgitation. Relying solely on visual assessments may result in misclassifying approximately half or more of moderate to severe regurgitations, which could delay heart valve interventions.

## Conclusion

This study proposes a balanced approach to improve patient management and outcomes in valvular heart disease. The approach involves integrating colour Doppler as a preliminary step followed by detailed quantitative analysis. While visual estimations may be sufficient for identifying mild cases, the study confirms the necessity of quantitative methods for moderate to severe regurgitations.

### Limitations

This study has several limitations. The use of pre-selected TTE loops may not accurately represent the full spectrum of clinical scenarios encountered in clinical practice. This approach may bias the participants towards certain types of valvular regurgitation or severities, thereby limiting the applicability of the findings to a real-world setting. Furthermore, it is important to note that the study’s use of digital assessments through an online survey may not fully capture the diagnostic process in a clinical setting. Factors such as the patient’s clinical status and real-time image acquisition may contribute to the diagnostic process in a clinical setting.

Furthermore, the negative association found between attending a hands-on course and assessment accuracy suggests that the quality and content of these courses may vary, affecting their effectiveness in improving echocardiographic skills. Additionally, the study population was limited to participants from three European countries, potentially limiting the generalizability of the findings to other regions with different training standards and healthcare systems.

## Electronic supplementary material

Below is the link to the electronic supplementary material.

Supplementary Material 1

Supplementary Material 2

Supplementary Material 3

Supplementary Material 4

## Data Availability

No datasets were generated or analysed during the current study.

## Abbreviations

3D: 3-dimensional
4D: 4-dimensional
AR: aortic valve regurgitation
CMR: cardiac magnetic resonance
CW: continuous wave
ESC: European Society of Cardiology
EACVI: European Association of Cardiovascular Imaging
LVF: left ventricular function
MR: mitral valve regurgitation
PISA: proximal isovelocity surface area
TR: tricuspid valve regurgitation
TTE: transthoracic echocardiography

## Declarations

### Clinical trial number

This study does not qualify for a clinical trial number because it does not involve the testing of any intervention, treatment, or drug on human participants.
