# Supplementary material for: Visual grading of valvular regurgitation is inferior to measurement – results from the VIAVA-study (VIsual Assessment of VAlvular Regurgitation)
Source: Echo Res Pract. 2024 Nov 11;11:26. doi: 10.1186/s44156-024-00061-0 (PMC11552230; doi:10.1186/s44156-024-00061-0)
Supplement: Supplementary file 2 — Supplementary Material 2 [file 44156_2024_61_MOESM2_ESM.pdf]

# Supplemental Material

## Visual grading of valvular regurgitation is inferior to measurement – results from the VIAVA-study (VIsual Assessment of VAlvular Regurgitation).

Ozan Demirel[1], Paolo Di Stefano[1], Elke Boxhammer[1], Thomas Wuppinger[1], Christina Granitz[1], Björn Goebel[2], Uta C. Hoppe[1], Michael Lichtenauer[1], Moritz Mirna[1]

[1] Clinic of Internal Medicine II, Department of Cardiology, Paracelsus Medical University of Salzburg, Austria

[2] Heart Center of the Central Clinic Bad Berka, Department of Cardiology, Bad Berka, Germany

Suppl. Figure 1

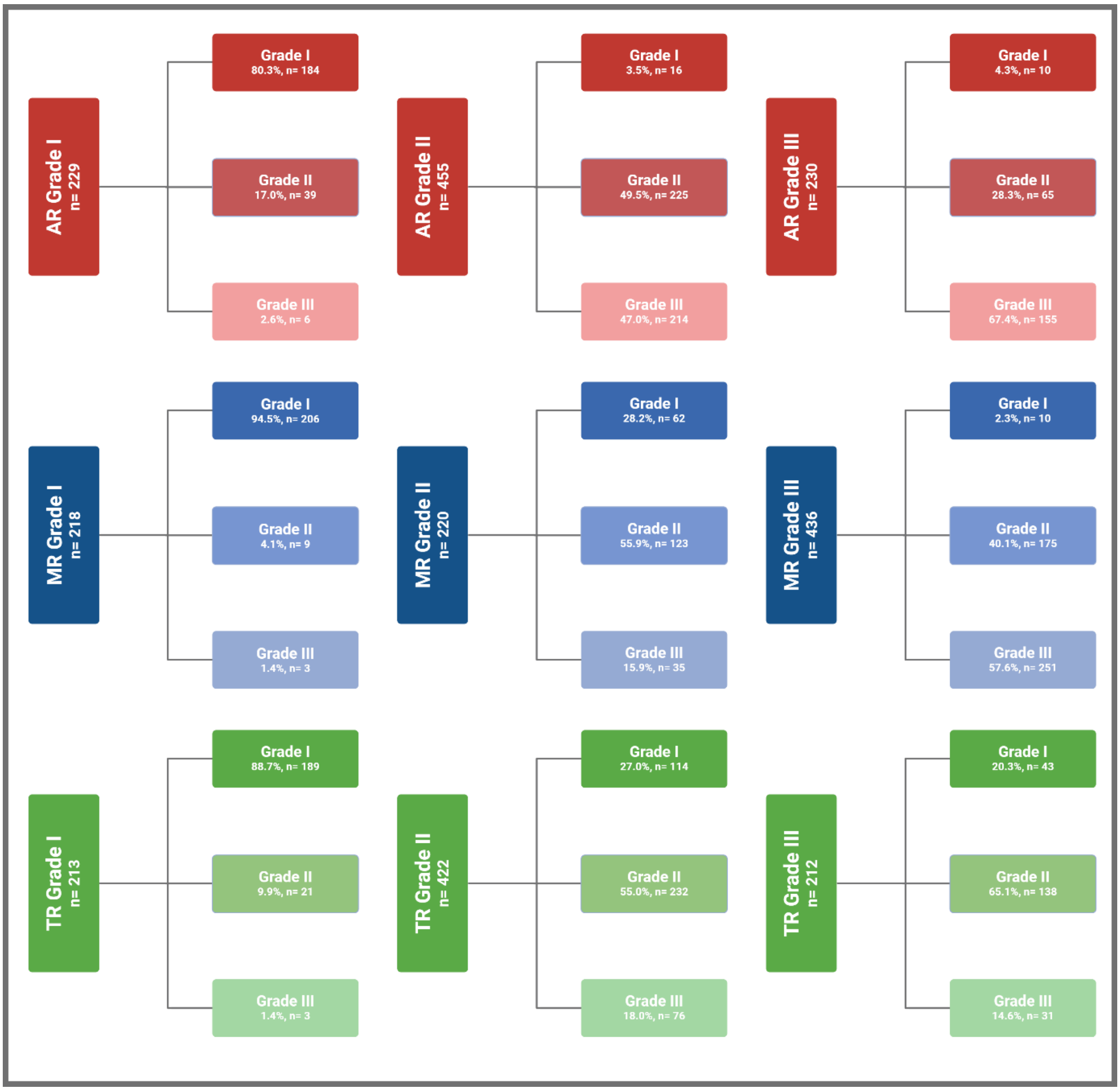


Visual assessments of each valvular regurgitation submitted by participants in relation to gradings attributed by the experts. Abbreviations: AR= aortic valve regurgitation, MR= mitral valve regurgitation, TR= tricuspid valve regurgitation. (Created using Biorender.com).

Suppl. Figure 2

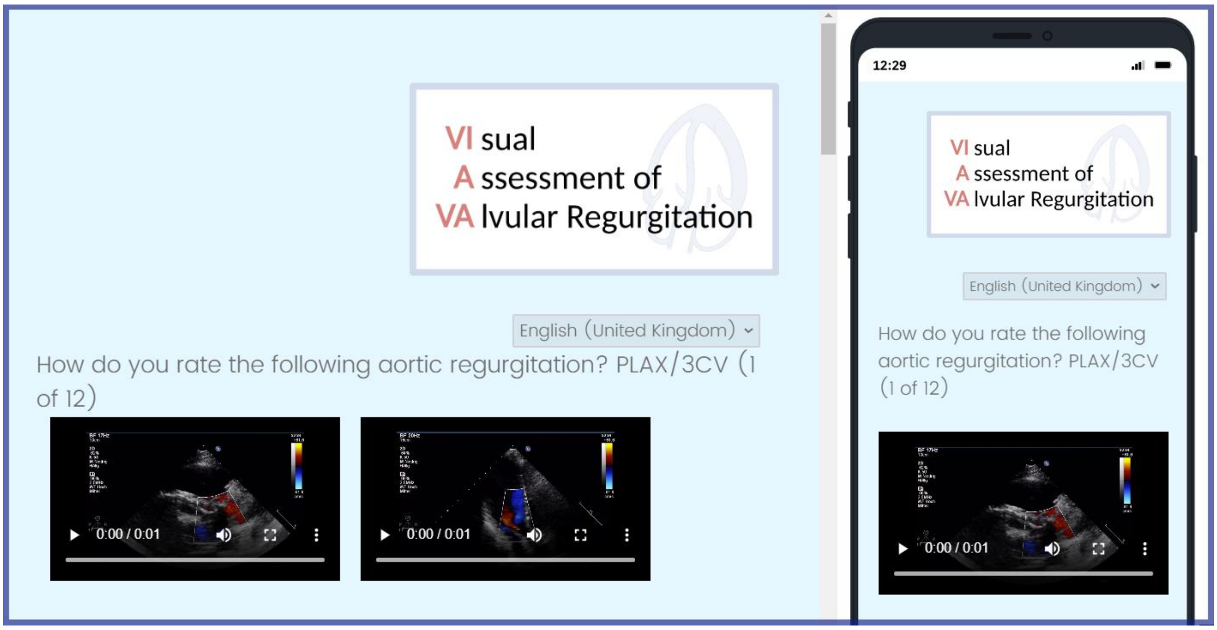


Computer and smartphone interface of the online survey in the Qualtrics CoreXM platform (Qualtrics, Provo, United States).

Suppl. Table 1

| | AR 1 | AR 2 | AR 3 | AR 4 |
|---|---|---|---|---|
| Colour flow Doppler Image | 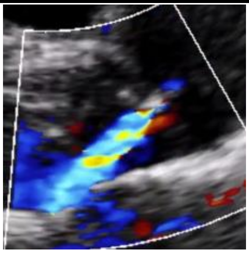 | 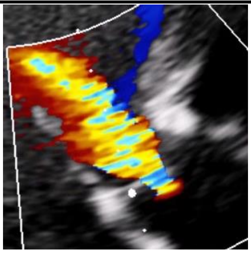 | 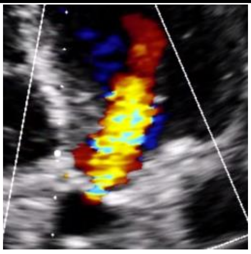 | 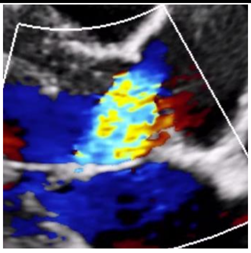 |
| VC (mm) | 3 | 4.4 | 4.6 | 9.4 |
| PHT (ms) | 914.4 | 554,2 | 643.3 | 555 |
| PISA-Radius (cm) | - | 0,61 | 0.58 | 1.59 |
| EROA (mm²) | - | 17 | 15 | 1.45 |
| Volume (ml) | - | 51.2 | 27.7 | 354.6 |
| prominent holodiastolic flow reversal in descending aorta | - | no | no | yes |
| Large flow convergence | no | no | no | yes |
| central Jet width of LVOT | < 25% | 0,41 | 0,48 | > 65% |

| | MR 1 | MR 2 | MR 3 | MR 4 |
|---|---|---|---|---|
| Colour flow Doppler Image | 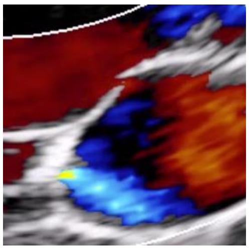 | 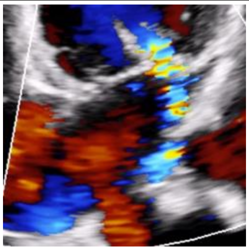 | 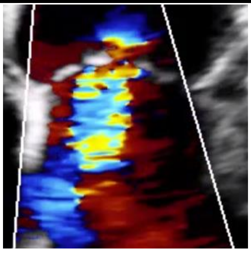 | 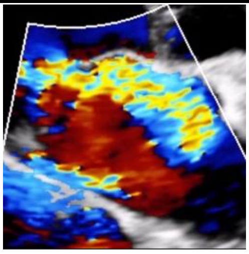 |
| VC (mm) | 3 | 5 | 7.4 | 9.8 |
| PISA-Radius (cm) | - | 0.9 | 1.18 | 1.61 |
| EROA (mm²) | - | 29 | 57 | 81 |
| Volume (ml) | - | 58.3 | 78 | 132.3 |

| | TR 1 | TR 2 | TR 3 | TR 4 |
|---|---|---|---|---|
| Colour flow Doppler Image | 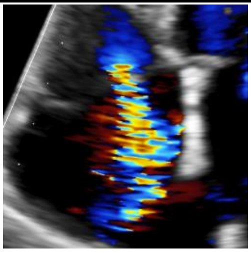 | 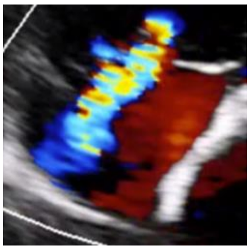 | 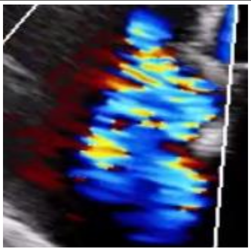 | 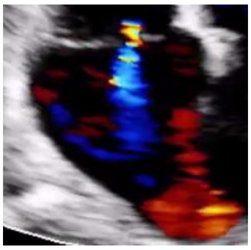 |
| VC (mm) | 4.5 | 6 | 7 | 3 |
| PISA-Radius (cm) | 0.46 | n/a | 0.76 | n/a |
| EROA (mm²) | 0.16 | n/a | 0.36 | n/a |
| Volume (ml) | 11.8 | n/a | 41.2 | n/a |
| TR Vmax (cm/s) | 329.6 | 377.4 | 309.5 | 230.9 |
| TR maxPG (mmHg) | 43.5 | 57.1 | 38.4 | 21.3 |

Measurements of valve regurgitations with colour flow Doppler images of the provided echoloops. *Abbreviations: AR= aortic regurgitation, MR= mitral regurgitation, TR= tricuspid regurgitation, VC= vena contracta, PHT= pressure half time, PISA= proximal isovelocity surface area, EROA= effective regurgitation orifice area, LVOT= left ventricle outflow tract, Vmax= maximal velocity, max PG= maximal pressure gradient, n/a = not available.*
