# Supplementary figures and images for: Visual grading of valvular regurgitation is inferior to measurement – results from the VIAVA-study (VIsual Assessment of VAlvular Regurgitation)
Source: Echo Res Pract. 2024 Nov 11;11:26. doi: 10.1186/s44156-024-00061-0 (PMC11552230; doi:10.1186/s44156-024-00061-0)

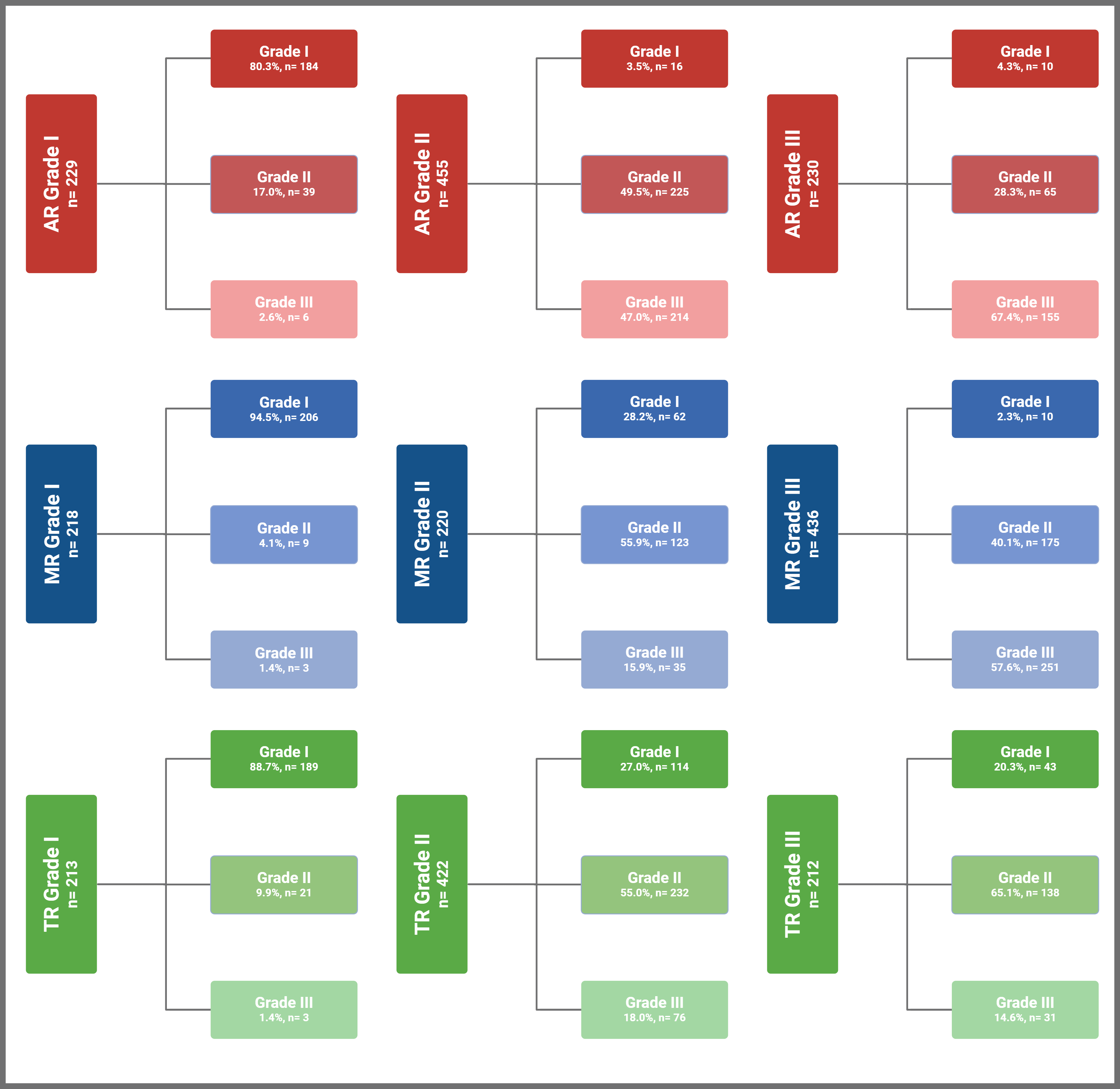

Supplement: Supplementary file 3 — Supplementary Material 3 [file 44156_2024_61_MOESM3_ESM.png]

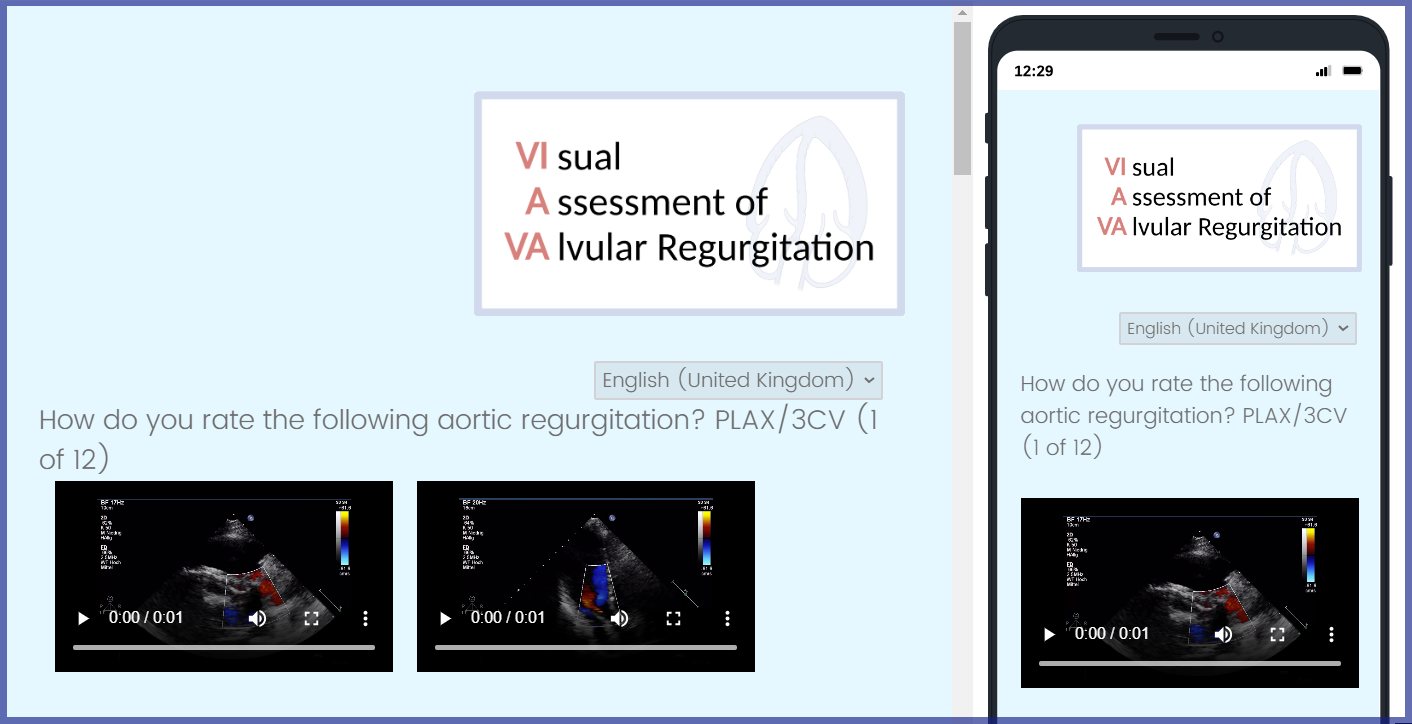

Supplement: Supplementary file 4 — Supplementary Material 4 [file 44156_2024_61_MOESM4_ESM.png]
